# CD44s-activated tPA/LRP1-NFκB pathway drives lamellipodia outgrowth in luminal-type breast cancer cells

**DOI:** 10.3389/fcell.2023.1224827

**Published:** 2023-09-28

**Authors:** Yaqi Qiu, Hui Wang, Qian Guo, Yiwen Liu, Yiqing He, Guoliang Zhang, Cuixia Yang, Yan Du, Feng Gao

**Affiliations:** ^1^ Department of Molecular Biology, Shanghai Sixth People’s Hospital Affiliated to Shanghai Jiao Tong University School of Medicine, Shanghai, China; ^2^ Department of Clinical Laboratory, Shanghai Sixth People’s Hospital Affiliated to Shanghai Jiao Tong University School of Medicine, Shanghai, China

**Keywords:** CD44, TPA, lamellipodia, luminal type breast cancer, LRP1

## Abstract

Some cancer cells migration and metastasis are characterized by the outgrowth of lamellipodia protrusions in which the underlying mechanism remains unclear. Evidence has confirmed that lamellipodia formation could be regulated by various adhesion molecules, such as CD44, and we previously reported that lamellipodia at the leading edge of luminal type breast cancer (BrCa) were enriched with high expression of CD44. In this study, we found that the overexpression of CD44s could promote lamellipodia formation in BrCa cells through inducing tissue type plasminogen activator (tPA) upregulation, which was achieved by PI3K/Akt signaling pathway activation. Moreover, we revealed that tPA could interact with LDL receptor related protein 1 (LRP1) to activate the downstream NFκB signaling pathway, which in turn facilitate lamellipodia formation. Notably, inhibition of the tPA/LRP1-NFkB signaling cascade could attenuate the CD44s-induced lamellipodia formation. Thus, our findings uncover a novel role of CD44s in driving lamellipodia outgrowth through tPA/LRP1-NFkB axis in luminal BrCa cells that may be helpful for seeking potential therapeutic targets.

## 1 Introduction

Breast cancer (BrCa) is the most common cancer in women and distant metastasis is a major obstacle to clinical treatment (Jemal et al., 2011). It is believed that cancer metastasis depends on the increasing capacity of migration and invasion of cancer cells which is usually characterized by the formation of cell-surface protrusions, including lamellipodia, filopodia, and invadopodia (Yamaguchi et al., 2005; Yilmaz and Christofori, 2009). These structures are F-actin enriched protrusions on the leading edges of moving cells that lead the cells to migrate or invade toward the external signals (Ridley et al., 2003; Yilmaz and Christofori, 2009). Until now, the underlying mechanism of cancer cell-membrane protrusions outgrowth is still poorly understood.

Reports showed that dynamic cell-surface protrusions were displayed in the leading subpopulation of collective migrating cells to sense the extracellular environments (Mayor and Etienne-Manneville, 2016). We previously found that CD44 high expressing cancer cells were distributed to the front edge during collective migration of luminal type BrCa (Yang et al., 2019). Meanwhile, others confirmed that luminal type BrCa mainly displayed lamellipodia rather than filopodia or invadopodia (Karamanou et al., 2020). In this paper, we ask for the connection between CD44 and lamellipodia formation in leading cells. Previous report has demonstrated that CD44 was found densely labeling the structure of lamellipodia in human lung carcinoma A549 cells (Kim et al., 2011). Moreover, CD44 was also reported to interact with some F-actin-binding proteins and cooperatively regulate actin cytoskeletal, whose rearrangement participated in the lamellipodia formation (Machesky, 2008; Chen et al., 2018). Hence, it is reasonable to speculate that CD44 may regulate the lamellipodia outgrowth.

As a cell-membrane receptor, activated CD44 is capable of triggering intracellular signaling pathways like PI3K/Akt, NF-κB, ras-MAPK, and c-jun to facilitate the transcription of target genes (Bourguignon et al., 2009; Chen and Bourguignon, 2014; Ouhtit et al., 2018). Urokinase plasminogen activator (uPA), a plasminogen activator that can convert the plasminogen into the its active form plasmin, is one of the target genes (Kobayashi et al., 2002; Montgomery et al., 2012; Mekkawy et al., 2014). A previous study has reported that hyaluronan (HA)-induced promotion of CD44 signaling potentiated the invasive process of breast cancer cells through regulating the expression of uPA (Montgomery et al., 2012). Moreover, it has been reported that another plasminogen activator, tissue type plasminogen activator (tPA) was significantly associated with tumor migration due to its activation of membrane receptors, like LRP1, Annexin2, and EGFR (Díaz et al., 2004; Ortiz-Zapater et al., 2007; Roda et al., 2009; Salama et al., 2019). Additionally, this cytokine-like function of tPA further triggers signaling pathways such as NFκB, MEK-ERK, and p-38 MAPK, contributing to a series of cellular activities *in vivo* and *in vitro* (Zhang et al., 2007; Lin et al., 2015; Salama et al., 2019). However, whether tPA could promote lamellipodia formation or function together with CD44 has not been reported so far.

The present study aims to investigate how CD44 regulates lamellipodia outgrowth in luminal type BrCa and to explore its underlying mechanism. First, we performed transcriptional analysis on CD44s overexpression BrCa cells to search for the potential genes in regulating CD44s-dependent lamellipodia outgrowth. We identified that tPA was significantly upregulated in response to CD44s overexpression through PI3K/Akt signaling pathway. Notably, the knockdown of tPA could dramatically suppress the enhanced formation of lamellipodia stimulated by CD44s. Next, we inhibited the membrane receptors of tPA and found that LRP1 was the main mediator of CD44s-tPA induced lamellipodia extension by activating NFκB signaling pathway. These findings demonstrated a new role of CD44s in lamellipodia outgrowth by activating tPA/LRP1- NFκB axis, which might be helpful to luminal type BrCa target therapy.

## 2 Materials and methods

### 2.1 Cell culture and reagents

The human BrCa cell line MCF7 and T47D were purchased from the American Type Culture Collection (ATCC). MCF7 cells were cultured in MEM supplemented with 10% fetal calf serum, 10 μg/mL insulin, 100 U/mL penicillin, and 100 mg/mL streptomycin. T47D cells were cultured in RPMI-1640 supplemented with 10% fetal calf serum, 7.5 μg/mL, 100 U/mL penicillin, and 100 mg/mL streptomycin. All cells were grown to 80% confluency for the experiments. All cell lines were cultured at 37°C in humidified air with 5% CO2 and 95% air. Primary antibodies against CD44 (ab189524, Abcam, Cambridge, UK), tPA (10147-1-AP, Proteintech, Chicago, United States), cortactin (ab81208, Abcam), p-Akt (4,060, CST, Danvers, United States), Akt (4,691, CST), β-actin (3,700, CST), LDL receptor related protein 1 (LRP1, BM4098, Boster, Wuhan, China), p-p65 (3,033, CST), p65 (8,242, CST), and inhibitors LY294002 (S1105, Selleck, Texas, United States), receptor associated protein (RAP, 11,100-H08H, Sino Biological, Beijing, China), LCKLSL (HY-P2333A, MCE, New Jersey, United States), AG1478 (153,436-53-4, MCE), BAY 11-7082 (HY-13453, MCE) were used.

### 2.2 siRNA, plasmids and lentivirus

The small interference RNA (siRNA) constructs targeting human tPA and human LRP1 expression were designed and synthesized by RiboBio company (Guangzhou, China), and the transfection was performed with riboFECTTM according to the manufacturer’s protocol. The siRNA sequences were listed as follows: tissue type plasminogen activator (tPA) siRNA (5′-CCC​UCU​CUU​CAU​UGC​AUC​CAU-3′), LRP1 siRNA (5′- GCU​CAU​CUC​GGG​CAU​GAU​U-3′), and scramble negative control siRNA (5′-UUC​UCC​GAA​CGU​GUC​ACG​U-3′). The human CD44s (NM_001001391) was constructed by PCR-based amplification and then cloned into the Ubi-MCS-SV40-IRES-puromycin vector system (Genechem Company, Shanghai, China). Next, cells were infected with concentrated lentivirus according to the manufacturer’s instructions and after infection, puromycin (2 μg/mL, Sigma Aldrich, St. Louis, MO) was used to select stably transduced cells over a 15-day period. Finally, the overexpression efficiency was verified by western blotting assay.

### 2.3 RNA sequencing and data analysis

RNA sequencing analysis of MCF7^vector^ and MCF7^CD44s^ cells was performed using an Illumina Novaseq 6000 instrument at the OE Biotech Co., Ltd. (Shanghai, China). Fragments Per kb Per Million Reads (FPKM) of each gene was calculated using Cufflinks, and the read counts of each gene were obtained by HTSeq-count. Differential expression analysis was performed using the DESeq (2012) R package. We utilized a fold change >1.5 with *p*-value <0.05 as the threshold for significantly differential expression. Gene ontology (GO) enrichment and KEGG pathway enrichment analysis of differentially expressed genes were performed respectively using R based on the hypergeometric distribution. Gene set enrichment analysis (GSEA; https://www.broadinstitute.org/gsea/index.jsp) was performed to analyze whether a set of genes show statistically significant differences between CD44s overexpression and the control groups.

### 2.4 Quantitative real-time PCR (qPCR)

Total RNA was extracted from cultured cells using RNAiso Plus (9108, Takara Bio, Shiga, Japan) according to the manufacturer’s instructions. The RNA concentration was measured using NanoDrop system (Thermo Fisher Scientific Inc. Waltham, MA). Then, 1 µg RNA was reverse transcribed using the PrimeScript™ RT Reagent kit with gDNA Eraser (RR047Q, Takara Bio). qPCR assays were performed with SYBR Green mix (RR820A, Takara Bio) according to the manufacturer’s protocol. The qPCR conditions included: initial denaturation at 95°C for 30 s, followed by 40 cycles of denaturation at 95°C for 5 s annealing and extension at 60°C for 34 s. All qPCR values of each gene were normalized against that of GAPDH. The relative gene expression was calculated using the 2 -ΔΔCT method.

The primer sequences for qPCR are listed in Supplementary Table S1.

### 2.5 Western blotting

RIPA buffer (P0013B, Beyotime, Shanghai, China) was used for protein extraction. After the total protein concentration was determined by a bicinchoninic acid protein assay kit (BCA1, Sigma Aldrich, St. Louis, MO). 20 μg protein samples were separated by 7.5% SDS polyacrylamide gels and transferred onto PVDF membranes (ISEQ00010, Millipore, Massachusetts, United States). The membrane was blocked with 5% non-fat milk in TBST for 1 h and incubated with the indicated antibody at 4°C overnight. Then HRP-conjugated secondary anti bodies (1:5000) were added. Bands were subsequently visualized using the enhanced plus chemiluminescence assay (SQ201, Epizyme, Shanghai, China). Measurement of the bands was conducted on an ImageQuant LAS 4000 mini.

### 2.6 Wound-healing assay

Cells were grown to 100% confluency in MEM supplemented with 10% FBS. A sterile pipette tip was used to make a straight line wound on the confluent cell culture. After washing the plate for 3 times to remove the mechanically detached cells and to expose the “wound,” the remaining cells were incubated for 72 h in MEM plus 1% FBS. At the indicated time points, the cell culture was photographed under a microscope.

### 2.7 Immunofluorescence

For immunofluorescence, cells were fixed in 4% paraformaldehyde, permeabilized with 0.2% triton X-100/phosphate-buffered saline (PBS), and blocked in 5% BSA. Cells were incubated with primary antibodies overnight at 4°C in primary antibody dilution buffer (P0256, Beyotime) and then with secondary antibodies conjugated with 594 (ab150077, Abcam) for 1 h at room temperature. For F-actin, cells were stained with Phalloidin-iFluor 647 reagent (ab176759, Abcam) for 1.5 h. Images were analyzed under confocal microscopy (Nikon A1, Tokyo, Japan).

### 2.8 Statistical analysis

Statistical analysis was performed using GraphPad Prism 9.0.0 (GraphPad Software, Inc.). Data are presented the as mean ± standard deviation (SD). The statistically significant of differences between the two groups were determined with an unpaired Student’s t-test. The significance of differences among groups was determined by one-way ANOVA, *t*-test or Fisher’s exact test. Statistical significance was defined as *p* < 0.05.

## 3 Results

### 3.1 Identification of tPA as a target of CD44s in luminal type breast cancer cells

**FIGURE 1:**
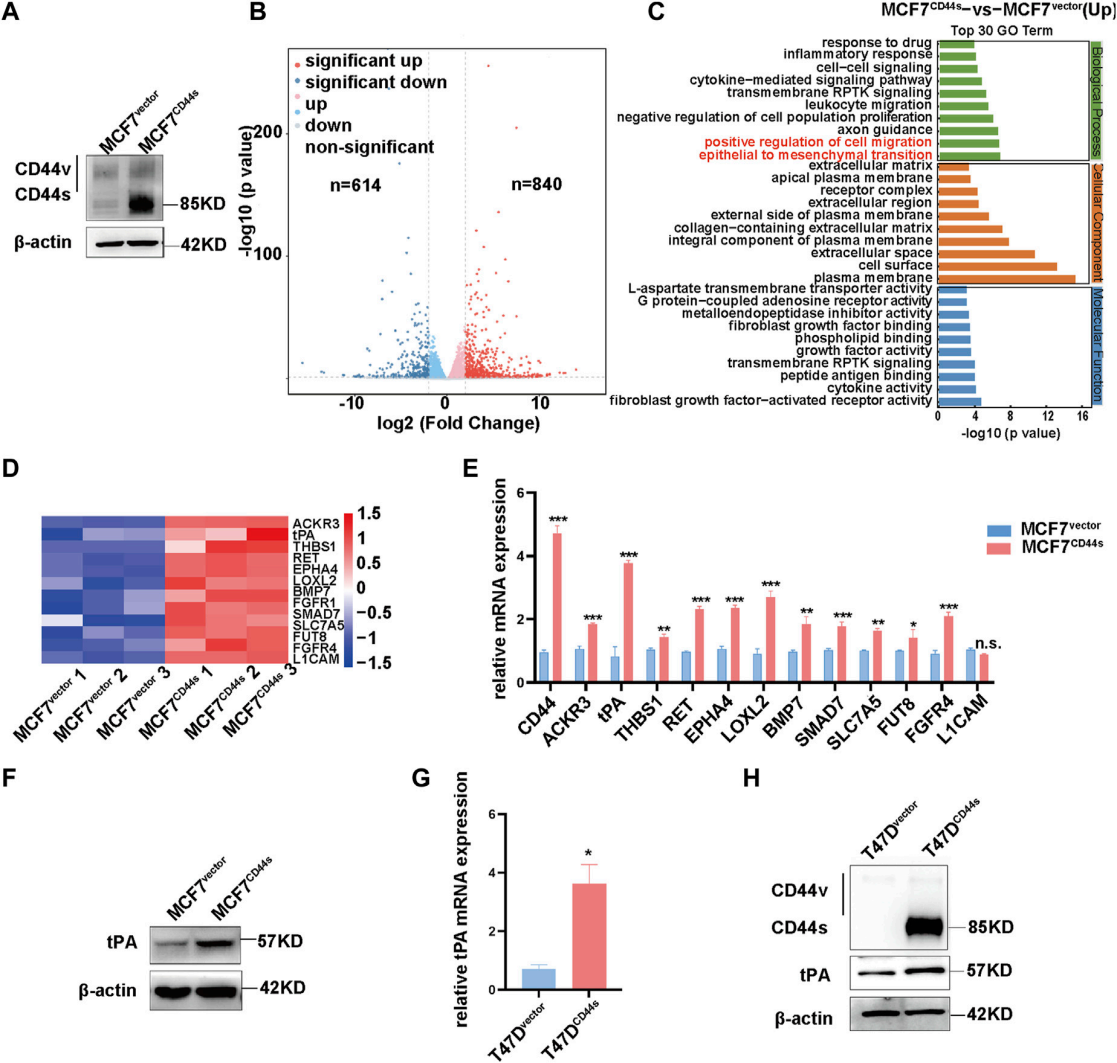
Identification of tPA as a target of CD44s in luminal type breast cancer cells **(A)** Overexpression efficiency of CD44s was evaluated by western blotting. **(B)** Volcano plot of log2 fold changes vs. -log10 *p*-value shows transcriptional differences between MCF7^vector^ and MCF7^CD44s^ overexpression cells. Vertical lines represent the 1.5-fold change cut-off and the horizontal lines indicate the 0.05 *p*-value cut-off. Up- and downregulated genes are highlighted in red and blue, respectively. **(C)** Gene ontology (GO) analysis of upregulated differentially expressed genes between MCF7^vector^ and MCF7^CD44s^ groups. **(D)** Heatmap shows expression profiles of the cancer cell migration and invasion related genes in MCF7^CD44s^ compared with MCF7^vector^. Red colors indicate upregulation. **(E)** Expression validation of candidate genes from heatmap by qPCR. The means ± SD of relative fold changes from triplicate experiments were plotted. GAPDH was used as the control. The *p* values were calculated by un-paired Student’s t-test. **(F)** Analysis of tPA expression by western blot in CD44s overexpression cells. **(G)** Analysis of tPA mRNA by qPCR in CD44s overexpression T47D cells. **(H)** Analysis of CD44s and tPA expression by western blot in CD44s overexpression T47D cells. *p < 0.05, **p < 0.01, ***p < 0.001.

CD44 activation triggers actin cytoskeleton reorganization to form lamellipodia, but the underlying mechanism has not been fully understood. To further identify the downstream target of CD44-dependent lamellipodia formation during luminal type BrCa progression, we firstly established a stable cell line by transfection of the CD44 standard form (CD44s) cDNA into MCF7 cells which initially express negligible CD44s (Figure 1A). Subsequently, we used RNA-sequencing (RNA-seq) to compare mRNA expression profiles between the MCF7^vector^ and MCF7^CD44s^ cells. As showed in Figure 1B, differential expression analysis (fold change >1.5, *p* < 0.05) highlighted 840 and 614 genes significantly up- and downregulated between control and CD44s overexpression groups. Moreover, functional annotation revealed that genes upregulated in CD44s overexpression cells were significantly enriched for positive regulation of cell migration and epithelial to mesenchymal transition (EMT) (Figure 1C), among which 13 genes were positively related to cancer cell migration or invasion (Figure 1D). Using qPCR, we confirmed that the expression of these genes was higher in MCF7^CD44s^ than in MCF7^vector^ cells (Figure 1E). Of note, tPA, one of the plasminogen activators, was the most robustly upregulated gene upon CD44s overexpression in MCF7 cells. Consistently, the protein level of tPA was significantly increased in MCF7^CD44s^ cells (Figure 1F). To further verify the regulation of tPA by CD44s in other luminal type BrCa cells, we established T47D cells stably overexpressing CD44s (Figure 1H). In line with the results in MCF7 cells, both mRNA and protein of tPA significantly elevated in T47D^CD44s^ cells (Figures 1G, H). Taken together, these results suggested that tPA might be the potential target of CD44s-induced lamellipodia outgrowth in luminal type BrCa.

### 3.2 tPA is involved in CD44s-dependent lamellipodia outgrowth of luminal type breast cancer cells

**FIGURE 2:**
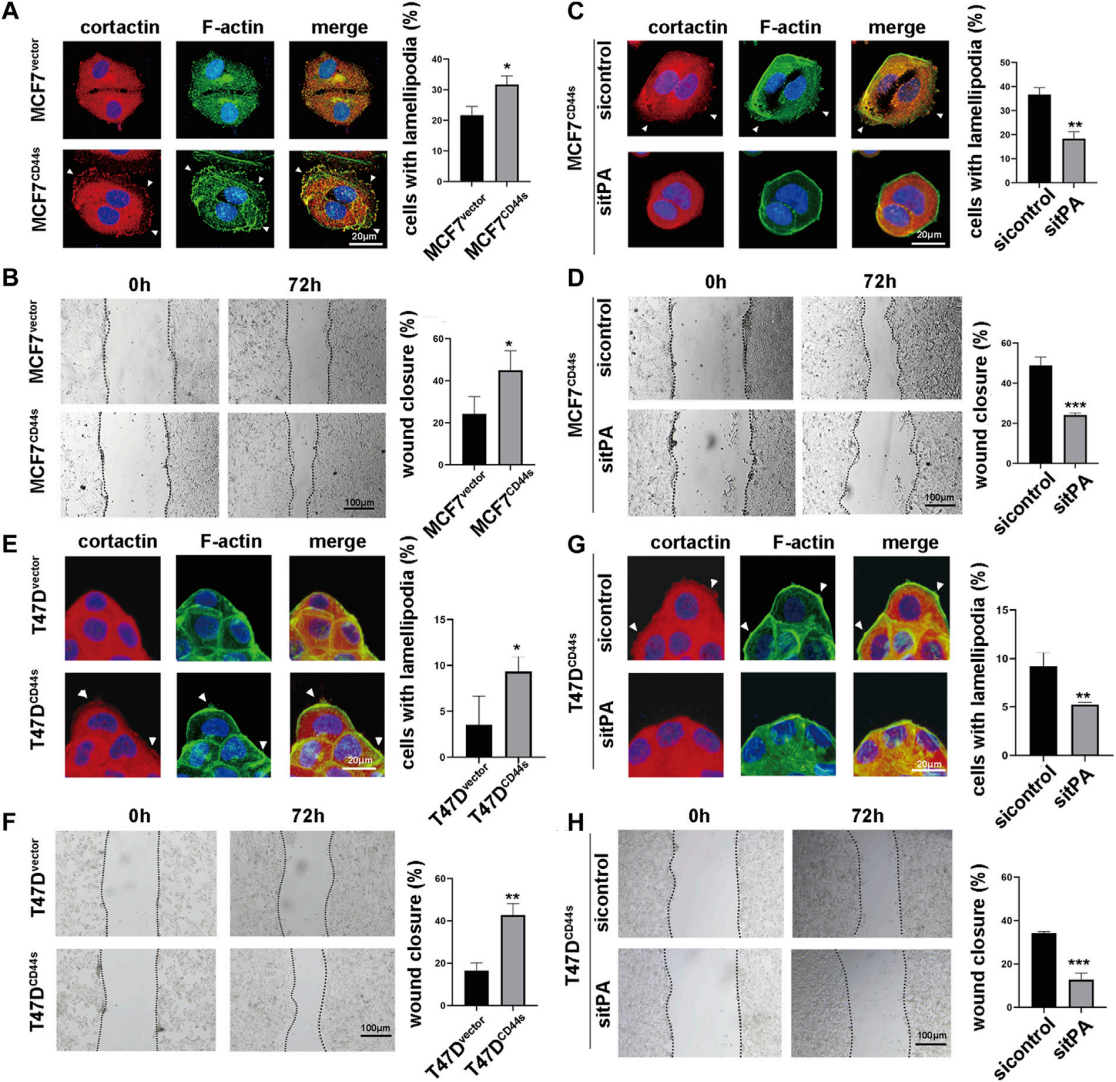
tPA is involved in CD44s-dependent lamellipodia outgrowth of luminal type breast cancer cells. **(A)** The distribution patterns of cortactin (red) and F-actin (green) demonstrated by immunofluorescence staining in MCF7^vector^ and MCF7^CD44s^ cells. The elongated lamellipodia are highlighted by the arrows. The proportion of cells with lamellipodia in MCF7^vector^ and MCF7^CD44s^ groups were calculated from triplicate independent experiments, means ± SD from triplicate experiments were plotted. **(B)** Representative images and quantitative analysis of migration assay showing the wound closure rate of MCF7^vector^ and MCF7^CD44s^ cells. The means ± SD of wound closure rates from triplicate experiments were plotted. **(C)** The distribution patterns of cortactin (red) and F-actin (green) demonstrated by immunofluorescence staining in control and tPA-knockdown MCF7^CD44s^ cells. The elongated lamellipodia are highlighted by the arrows. The proportion of cells with lamellipodia in control and tPA-knockdown groups were calculated from triplicate independent experiments. Means ± SD from triplicate experiments were plotted. **(D)** Representative images and quantitative analysis of migration assay showing the wound closure rate of control and tPA-knockdown MCF7^CD44s^ cells. **(E)** The distribution patterns of cortactin (red) and F-actin (green) demonstrated by immunofluorescence staining in T47D^vector^ and T47D^CD44s^ cells. The proportion of cells with lamellipodia in T47D^vector^ and T47D^CD44s^ groups were calculated from triplicate independent experiments, means ± SD from triplicate experiments were plotted. **(F)** Representative images and quantitative analysis of migration assay showing the wound closure rate of T47D^vector^ and T47D^CD44s^ cells. The means ± SD of wound closure rates from triplicate experiments were plotted. **(G)** The distribution patterns of cortactin (red) and F-actin (green) demonstrated by immunofluorescence staining in control and tPA-knockdown T47D^CD44s^ cells. The proportion of cells with lamellipodia in control and tPA-knockdown groups were calculated from triplicate independent experiments. Means ± SD from triplicate experiments were plotted. **(H)** Representative images and quantitative analysis of migration assay showing the wound closure rate of control and tPA-knockdown T47D^CD44s^ cells. The means ± SD of wound closure rates from triplicate experiments were plotted. n. s. Indicates no significant, *p < 0.05, **p < 0.01, ***p < 0.001.

To dissect the role of CD44s in lamellipodia outgrowth in luminal type BrCa, we next investigate whether CD44s overexpression affects lamellipodia protrusions. Given that lamellipodia was characterized by enhanced migration ability and rearrangement of cortactin and F-actin, wound-healing assay and immunofluorescence assay were performed. The cells were seeded on the coverslips for 72 h to evaluate their lamellipodia formation abilities, because the time course of lamellipodia formation showed that MCF7^CD44s^ and T47D^CD44s^ displayed the most noticeable lamellipodia at this time point (Supplementary Figure S1). As shown in Figure 2A, CD44s overexpression in MCF7 cell line stimulated the migration of cells across the wound space, while no significant difference in proliferation rate was observed between these two groups in the same incubation condition (Supplementary Figure S2), which confirming the role of CD44s in cell migration. Moreover, CD44s overexpressed cells displayed more abundant lamellipodia when compared to the control group (Figure 2B). To further verify the effect of CD44s on lamellipodia formation in luminal type BrCa cells, we performed the loss-of-function experiment by knocking down CD44s in CD44s overexpressing MCF7 cells. As shown in Supplementary Figure S3, the knockdown of CD44s attenuated the promoted proportion of lamellipodia positive cells mediated by CD44s. By contrast, in CD44s highly expressed basal-like BrCa cells, such as BT-549 and MDA-MB-231, CD44s knockout could not change their lamellipodia formation abilities (Supplementary Figure S4), indicating that the observed effects of CD44s in lamellipodia formation were more important to luminal type BrCa cells. Subsequently, we attempted to explore the contribution of tPA to CD44s-induced lamellipodia formation by knocking down tPA. Our result demonstrated that the knockdown of tPA attenuated the elevated migration and proportion of lamellipodia positive cells mediated by CD44s in MCF7 cells while no significant difference was observed when tPA was knockdown in MCF7^vector^ cells (Figures 2C, D; Supplementary Figure S5). These phenomena were then recapitulated in another luminal type BrCa cell line T47D (Figures 2E–H). Collectively, our results identified a novel CD44s-tPA pathway that played a crucial role in regulating lamellipodia extension in luminal type BrCa cells.

### 3.3 CD44s induces transcriptional upregulation of tPA through PI3K/AKT signaling pathway

**FIGURE 3:**
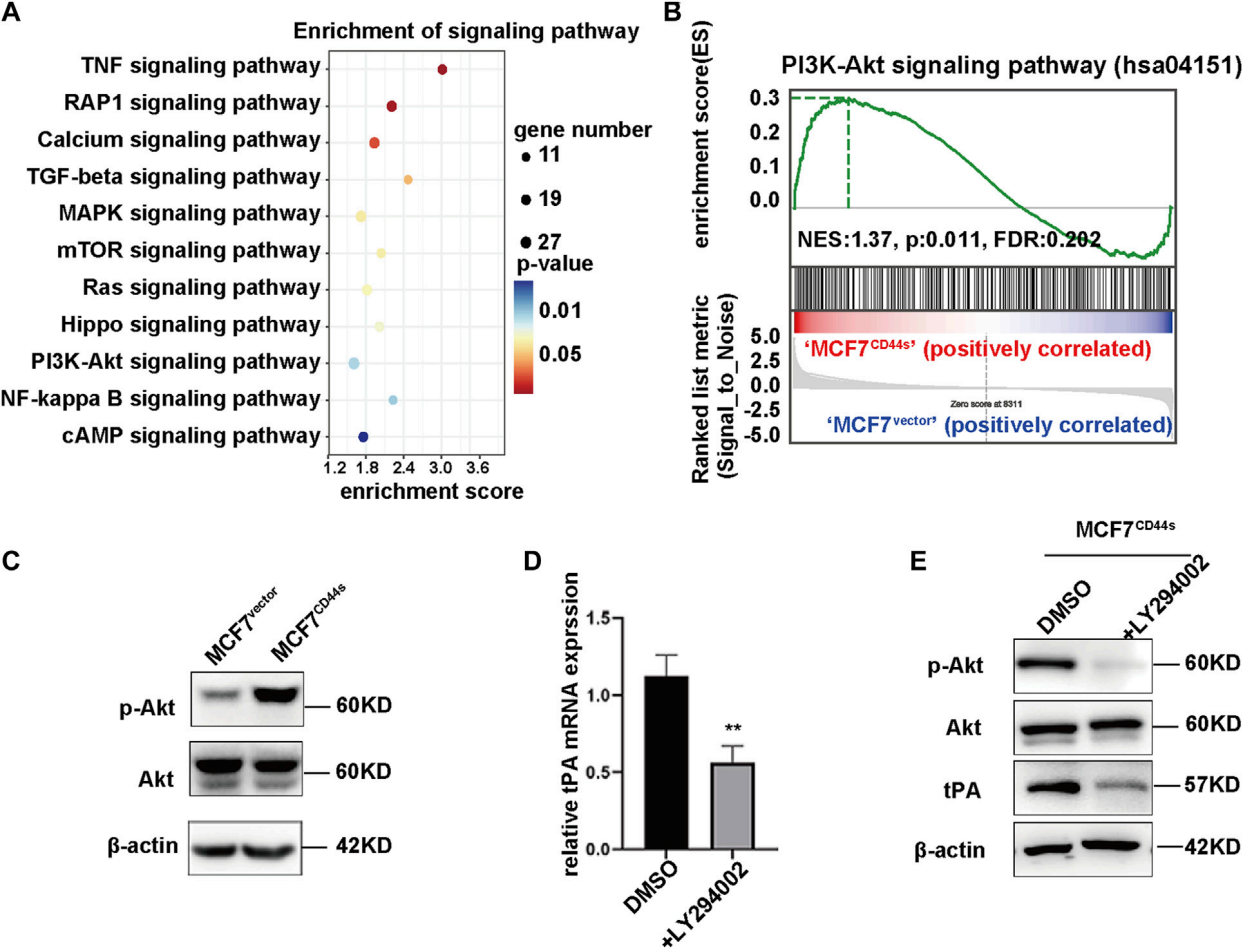
CD44s induces transcriptional regulation of tPA through PI3K/AKT signaling pathway. **(A)** KEGG analysis of activated signaling pathways in MCF7^CD44s^ group compared with MCF7^vector^ group. **(B)** Gene set enrichment analysis (GSEA) enrichment plots of the hallmark of PI3K/Akt gene sets in MCF7^CD44s^ compared with MCF7^vector^. FDR, false discovery rate. NES, normalized enrichment score. **(C)** Analysis of phosphorylation of Akt, total Akt protein levels in MCF7^vector^ and MCF7^CD44s^ cells by western blot. **(D)** Analysis of relative mRNA levels of tPA in control and PI3K inhibitor (LY294002, 20 µM) pretreated MCF7^CD44s^ cells by qPCR. **(E)** Analysis of phosphorylation of Akt, total Akt and tPA protein levels in control and PI3K inhibitor pretreated MCF7^CD44s^ cells by western blot.

As a membrane receptor, CD44 is capable of mediating multiple signaling pathways to regulate the transcription of its target genes. Thus, to gain insight into the mechanism by which CD44s induces tPA expression, we focused on the signaling pathways activated upon CD44s overexpression. By analysing Kyoto Encyclopedia of Genes and Genomes (KEGG), CD44s overexpression resulted in significantly activated 11 pathways (Figure 3A), among which PI3K/Akt cascade was reported to be able to induce tPA expression (Chang et al., 2006; Huang et al., 2006). Similarly, Gene Set Enrichment Analysis (GSEA) analysis confirmed a significant positive enrichment of PI3K/Akt cascade-engaged gene sets in MCF7^CD44s^ group compared to MCF7^vector^ cells (Figure 3B). Accordingly, we speculated that PI3K/Akt axis might be involved in the CD44s-induced upregulation of tPA. As expected, our western blot results showed that the phosphorylation level of Akt (Ser473) was markedly increased in CD44s overexpression cells (Figure 3C). Moreover, the PI3K/Akt pathway inhibitor LY294002 could impair the CD44s-triggered tPA upregulation both in mRNA and protein levels (Figures 3D, E). Collectively, we identified that CD44s might regulate tPA expression through PI3K/Akt axis to promote BrCa progression.

### 3.4 Membrane-receptor LRP1 is responsible for the stimulatory effect of CD44s-tPA on lamellipodia formation

**FIGURE 4:**
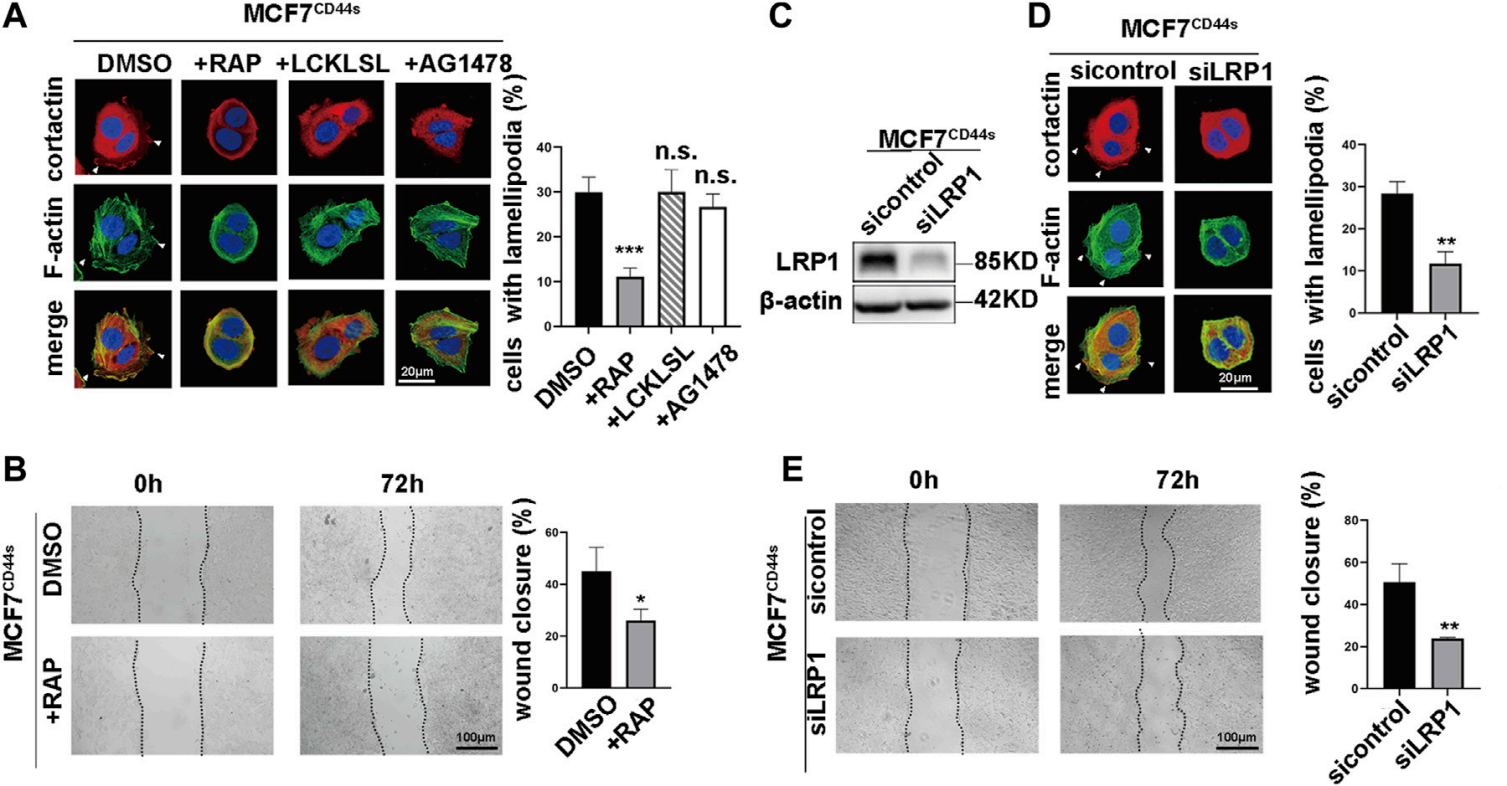
Membrane-receptor LRP1 is responsible for the stimulatory effect of CD44s-tPA on lamellipodia formation **(A)** The distribution patterns of cortactin (red) and F-actin (green) demonstrated by immunofluorescence staining in control and LRP1 inhibitor (RAP, 200 nM), Annexin A2 inhibitor (LCKLSL, 2.5 µM), and EGFR inhibitor (AG1478, 10 µM) pretreated CD44s-overexpressing MCF7 cells. The elongated lamellipodia are highlighted by the arrows. The proportion of cells with lamellipodia in control and RAP, LCKLSL, and AG1478 pretreated groups were calculated from triplicate independent experiments, means ± SD from triplicate experiments were plotted. **(B)** Representative images and quantitative analysis of migration assay showing the wound closure rate of control and RAP pretreated MCF7^CD44s^ cells. The means ± SD of wound closure rates from triplicate experiments were plotted. **(C)** Analysis of LRP1 expression in control and LRP1 knockdown MCF7CD44s cells. **(D)** Representative images and quantitative analysis of migration assay showing the wound closure rate of control and LRP1 knockdown MCF7^CD44s^ cells. The means ± SD of wound closure rates from triplicate experiments were plotted. **(E)** Representative images and quantitative analysis of migration assay showing the wound closure rate of control and LRP1 knockdown MCF7^CD44s^ cells. The means ± SD of wound closure rates from triplicate experiments were plotted. n. s. Indicates no significant, **p < 0.01, ***p < 0.001.

The aforementioned findings indicated that CD44s-tPA regulated lamellipodia formation. However, the mechanism linking CD44s-tPA to lamellipodia was still obscure. It was well accepted that tPA could act as a cytokine and transduce its signal through interacting with cell membrane receptors such as EGFR, Annexin A2 and LRP1 (Díaz et al., 2004; Ortiz-Zapater et al., 2007; Salama et al., 2019). To unravel the role of its membrane receptors in CD44s-tPA induced lamellipodia formation, we utilized their inhibitors, AG1748 (inhibitor for EGFR), LCKLSL (inhibitor for Annexin 2A) and RAP (receptor-associated protein, inhibitor for LRP1), respectively. Results showed that the migration rate and the proportion of lamellipodia positive cells elevated by CD44s overexpression were dramatically abrogated by RAP, a molecule that binds to LRP1 and blocks its interactions with all known ligands (Figures 4A, B), while no significant difference was observed in AG1748 or LCKLSL-pretreated groups. Furthermore, similar results were also obtained by knocking down LRP1, that is, the silence of LRP1 could attenuate the promotion of lamellipodia protrusions caused by CD44s overexpression (Figures 4D, E), while LRP1 knockdown in MCF7^vector^ cells did not affect lamellipodia formation (Supplementary Figure S5). Taken together, these data suggested that LRP1 was involved in CD44s-tPA-mediated lamellipodia induction in MCF7 cells.

### 3.5 tPA/LRP1 axis enhances the lamellipodia formation through NFκB signaling pathway

**FIGURE 5:**
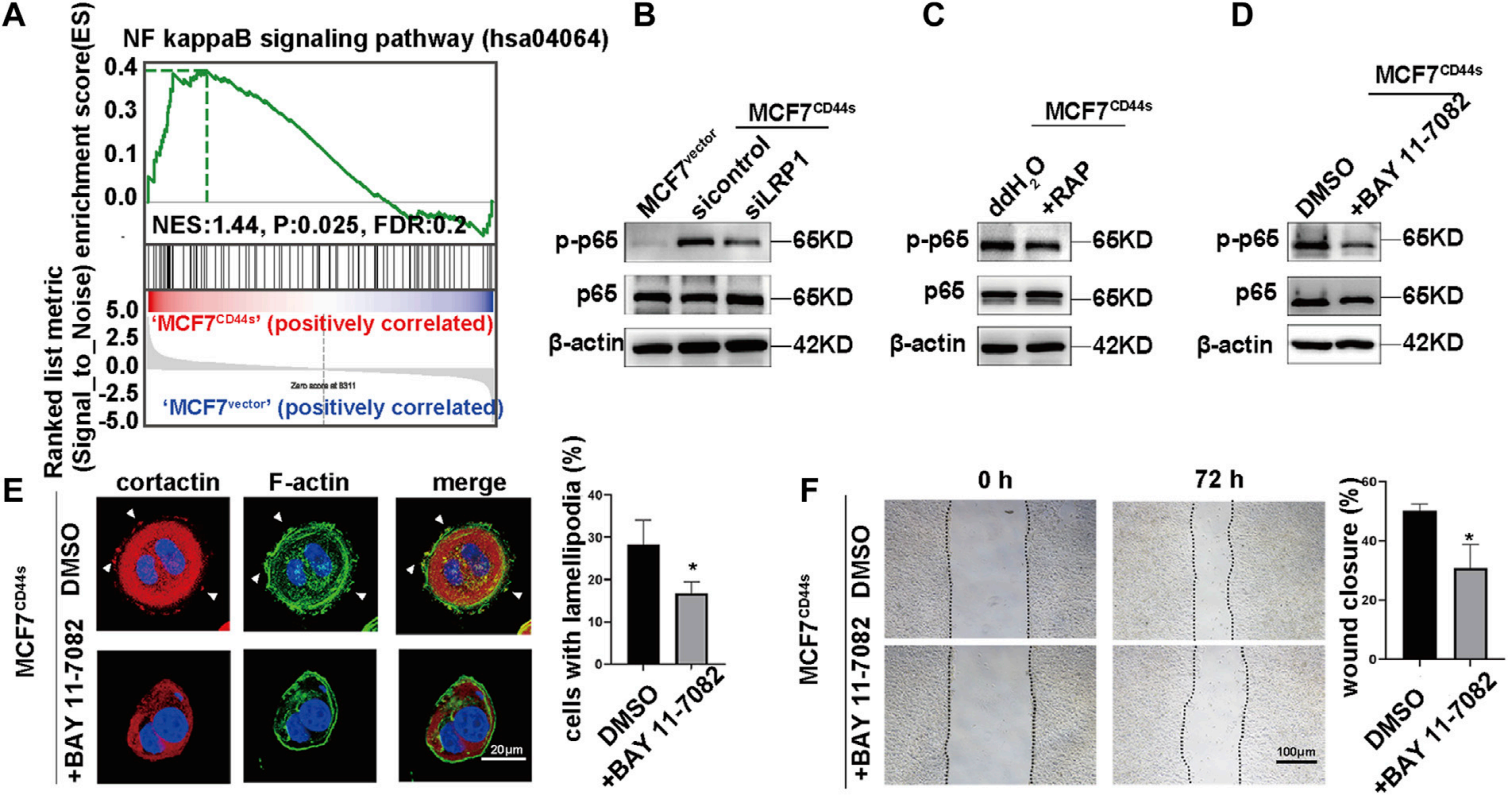
tPA/LRP1 axis enhances the lamellipodia formation through NFκB signaling pathway. **(A)** Gene set enrichment analysis (GSEA) enrichment plots of the hallmark of NFκB gene sets in MCF7^CD44s^ compared with MCF7^vector^ groups. **(B)** Analysis of phosphorylation of p65, total p65 protein levels in MCF7^vector^, MCF7^CD44s^, and LRP1 knockdown MCF7^CD44s^ cells by western blot. **(C)** Analysis of phosphorylation of p65, total p65 protein levels in control and RAP pretreated MCF7^CD44s^ cells by western blot. **(D)** Analysis of phosphorylation of p65, total p65 protein levels in control and NFκB pathway inhibitor (BAY 11-7082, 10 µM) pretreated MCF7^CD44s^ cells by western blot. **(E)** The distribution patterns of cortactin (red) and F-actin (green) demonstrated by immunofluorescence staining in control and BAY 11-7082 pretreated CD44s-overexpressing MCF7 cells. The elongated lamellipodia are highlighted by the arrows. The proportion of cells with lamellipodia in control and BAY 11-7082 pretreated groups were calculated from triplicate independent experiments, means ± SD from triplicate experiments were plotted. **(F)** Representative images and quantitative analysis of migration assay showing the wound closure rate of control and BAY 11-7082 pretreated MCF7^CD44s^ cells. The means ± SD of wound closure rates from triplicate experiments were plotted. *p < 0.05.

Next, we searched for the downstream cascades of CD44s-tPA/LRP1 that induce the lamellipodia formation. The result of KEGG and GSEA analysis of transcription data between control and CD44s overexpression MCF7 cells showed that NF-κB was one of the drown-stream signaling pathways of CD44s (Figure 3A; Figure 5A). Meantime, it has been hypothesized that tPA/LRP1 pathway was capable of activating NF-κB, which was reported to play a critical role in the formation of lamellipodia (Zhang et al., 2007; Guo et al., 2014). Thus, we presumed that the NFκB signaling pathway may be involved in oncogenic phenotype induced by CD44s-tPA/LRP1 axis. Given that phosphorylation of p65 (Ser536) is an indicator of NFκB pathway activation, we detected whether tPA/LRP1 could regulate the level of phosphorylated p65. As shown in Figures 5B, C, interference of LRP1 either by its inhibitor RAP or knocking down LRP1 could significantly suppress CD44s-induced p65 phosphorylation in MCF7 cells. To further confirm the contribution of NFκB signaling pathway to CD44s-tPA/LRP1-mediated lamellipodia outgrowth, we next investigate whether the inhibition of NFκB pathway affects the activated lamellipodia formation by CD44s overexpression. Our results indicated that the proportion of lamellipodia-positive cells was reduced when NFκB was inhibited (Figures 5D, E) and inhibition of NFκB had no effect on lamellipodia formation in MCF7^vector^ cells (Supplementary Figure S5). Consistent with this finding, CD44s-stimulated migration was also prevented by inactivation of NFκB (Figure 5F). These results suggested that NFκB signaling pathway was required for CD44s-tPA/LRP1 induced lamellipodia extension.

## 4 Discussion

Although CD44 has been extensively studied in context of cancer migration, few researches have been carried out to explore its role in regulating lamellipodia outgrowth in cancer migration. In this study, we identified that CD44s-tPA axis enhanced lamellipodia formation process *via* activating LRP1-NFκB signaling pathway in luminal type breast cancer (BrCa). Indeed, we have previously reported that CD44 was located at the leading edges of luminal type BrCa cells during collective migration and cells with high CD44 expression exhibited higher migration rate than those with low levels (Yang et al., 2019). Consistent with our findings, other studies have shown that CD44 is connected to the process of lamellipodia outgrowth in various types of cells. For example, knockout of CD44 in mouse bone marrow cells dramatically reduced the formation of lamellipodia induced by TNF-α and anti-CD44 antibody could abolish the HA-mediated lamellipodia information in mouse mammary epithelial cells (Oliferenko et al., 2000; Suzuki et al., 2002). To this end, we attempt to clarify the mechanism by which CD44 facilitates lamellipodia outgrowth in luminal type BrCa. First, we established two CD44s overexpression BrCa cell lines initially displaying low CD44s expression to observe whether CD44s had direct effect on cell lamellipodia formation. Unsurprisingly, overexpression of CD44s prompted the lamellipodia extension in BrCa cells.

Next, to ask for the CD44s involved mechanism in lamellipodia growing up, we compared the transcriptomic profiles between MCF7^vector^ and MCF7^CD44s^ cells. We identified that tissue type plasminogen activator (tPA) was a critical downstream target of CD44s. Moreover, we also identified that the downstream signaling pathway of CD44s was PI3K/Akt cascade. In fact, previous study has reported that tPA activation was associated with PI3K/Akt signaling (Huang et al., 2006), we then asked whether CD44s regulates tPA through PI3K/Akt pathway. Our results showed that the inhibition of PI3K/Akt axis decreased the CD44s-triggered tPA upregulation, implying that CD44s may regulate tPA by activating PI3K/Akt signaling pathway.

tPA is a serine protease that catalyzes the activation of plasminogen. Recently, some studies have connected tPA with cancer progression (Díaz et al., 2002; Roda et al., 2009; Salama et al., 2019; Noh et al., 2021). For instance, reports show that tPA modulates cancer cells proliferation, migration, invasion and angiogenesis through activation of tPA membrane receptors (Díaz et al., 2002; Díaz et al., 2004; Ortiz-Zapater et al., 2007). In the present study, we illustrated that knockdown of tPA hampered the lamellipodia outgrowth induced by CD44s in BrCa cells, indicating that CD44s-tPA may act as a novel axis to regulate cancer cell membrane protrusions. As studies have proved that tPA has to interact with its receptors to serve as a modulator, we next identify the receptor in lamellipodia outgrowth. Our experiments showed that the blocking of LDL receptor related protein 1 (LRP1) rather than inhibition of EGFR or Annexin A2, abolished the lamellipodia formation upregulated by CD44s-tPA in MCF7 cells. In fact, previous studies have demonstrated that EGFR and Annexin A2 were presumed to be involve in lamellipodia formation (den Hartigh et al., 1992; He et al., 2019; Filipenko and Waisman, 2001). We reasoned that it might be attributed to cell-type properties, especially the relatively low expression of EGFR and Annexin A2 in MCF7 cells (Sharma et al., 2010; Liao et al., 2019). Meanwhile, a previous study reported that LRP1 was involved in cancer cell migration and invasion, which was consistent with our findings (Tian et al., 2019). Anyway, our study suggested that LRP1 might be the mediator of tPA in luminal type BrCa cells lamellipodia outgrowth.

LRP1 is a multifunctional receptor implicated in both endocytosis and signaling pathways (Van Gool et al., 2015). Evidence shows that LRP1 is highly expressed in multiple types of cancers and is closely associated with cancer metastasis (Tian et al., 2019; Xue et al., 2022). As one of its ligands, tPA is reported to be able to induce LRP1 tyrosine phosphorylation, which in turn facilitates the subsequent signaling pathways (Hu et al., 2007). Our present study indicated that CD44s-tPA/LRP1 might be connected to an axis which can induce BrCa protrusion outgrowth. To further explore the target pathway responsible for such axis, we next analyzed the transcriptomic data and found that NFκB signaling was a candidate in CD44s-induced lamellipodia outgrowth. Our data showed that CD44s upregulation in MCF7 cells could activate tPA/LRP1-NFκB axis to facilitate lamellipodia formation, which was supported by the results that lamellipodia formation was attenuated after inhibiting NFκB. This result was in according with a previous study which showed that NFκB ablation could suppress the lamellipodia formation (Guo et al., 2014).

**FIGURE 6:**
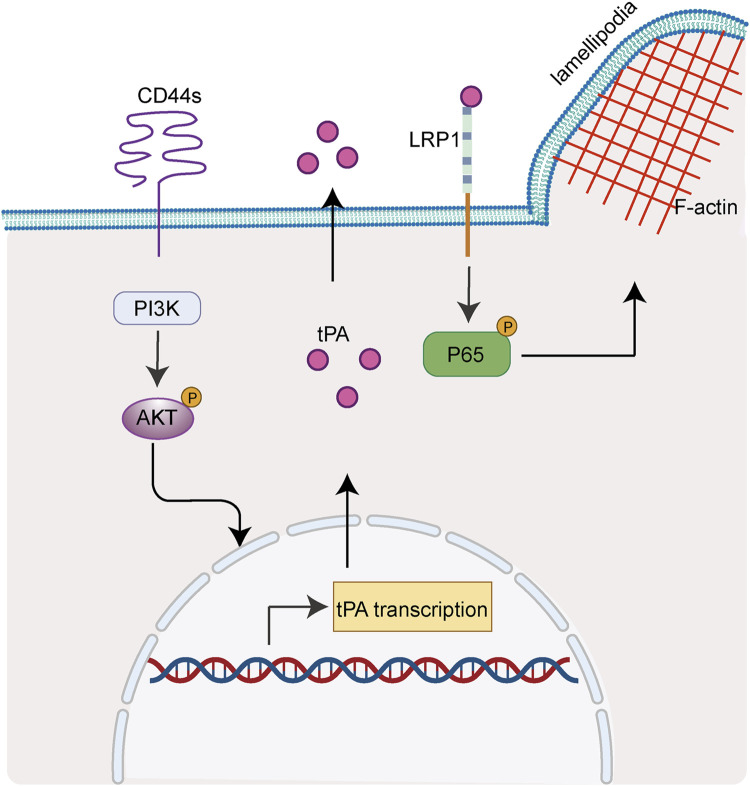
CD44s drives lamellipodia formation through PI3K-Akt-tPA/LRP1-NFκB cascade. Scheme summarizing the proposed mechanism by which CD44s drives lamellipodia formation in luminal type BrCa. We propose that CD44s upregulates tPA through PI3K/Akt signaling pathway, thus activating its membrane-receptor LRP1 and subsequently its drown-stream NFκB axis, ultimately leading to the outgrowth of lamellipodia.

Overall, the present study unveiled a novel role of CD44s in regulating lamellipodia outgrowth through tPA/LRP1-NFκB axis in luminal type BrCA cells (Figure 6). Our results may be helpful to the identification of new candidates for BrCa future targeted therapies.

## Data Availability

The datasets presented in this study can be found in online repositories. The names of the repository/repositories and accession number(s) can be found below: NCBI GEO and GSE233209 (https://www.ncbi.nlm.nih.gov/geo/query/acc.cgi?acc=GSE233209).
