# Supplementary material for: CD44s-activated tPA/LRP1-NFκB pathway drives lamellipodia outgrowth in luminal-type breast cancer cells
Source: Front Cell Dev Biol. 2023 Sep 28;11:1224827. doi: 10.3389/fcell.2023.1224827 (PMC10569302; doi:10.3389/fcell.2023.1224827)
Supplement: Supplementary file 1 [file DataSheet1.PDF]

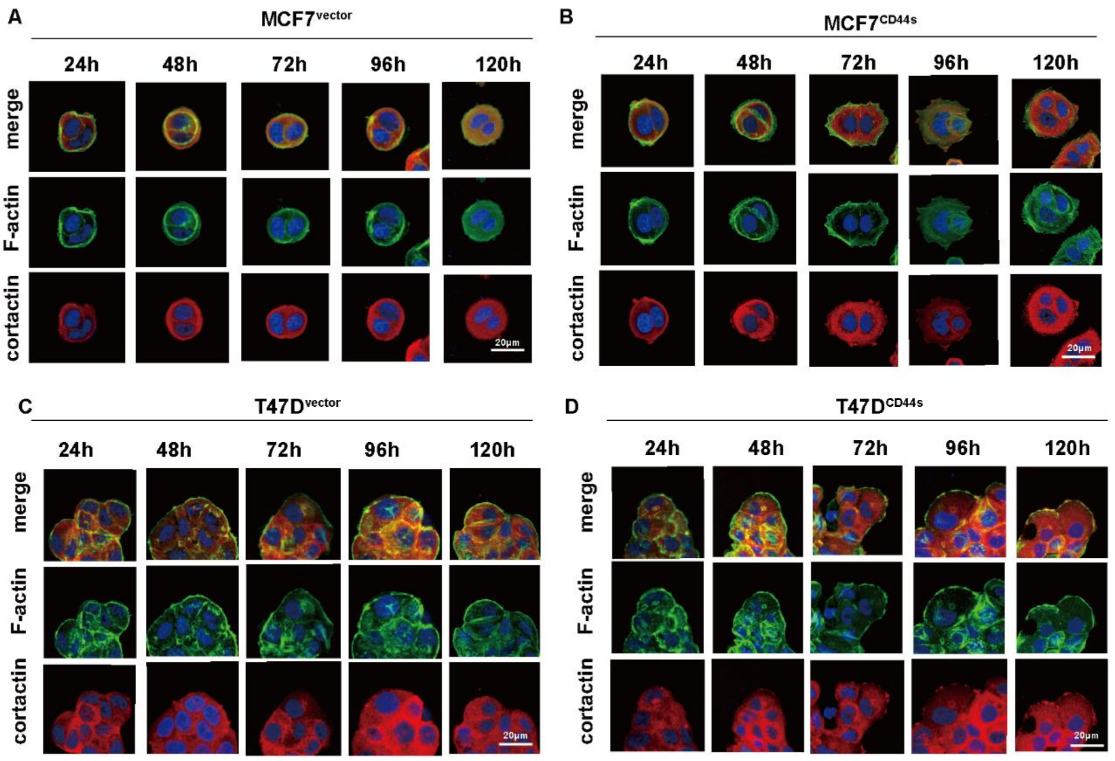

Fig. S1 The time course of lamellipodia formation in MCF7^{vector}, MCF7^{CD44s}, T47D^{vector}, and T47D^{CD44s} cells

(A)-(D) MCF7^{vector}, MCF7^{CD44s}, T47D^{vector}, and T47D ^{CD44s} cells were seeded on the coverslips for 24h, 48h, 72h, 96h, and 120h. The distribution patterns of cortactin (red) and F-actin (green) were demonstrated by immunofluorescence staining in these cells.

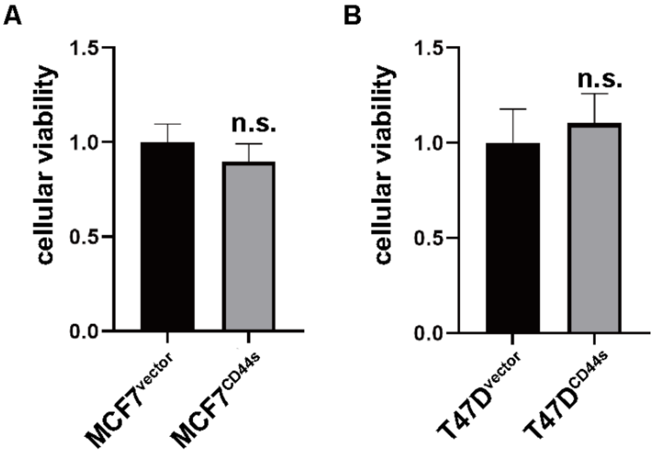

Fig. S2 The effect of CD44s on cell proliferation in MCF7 and T47D cells

(A) The cellular viability of MCF7^{vector} and MCF7^{CD44s} cells when incubated in medium plus 1% FBS for 72 h. (B) The cellular viability T47D^{vector}, and T47D^{CD44s} cells when incubated in medium plus 1 % FBS for 72 h.

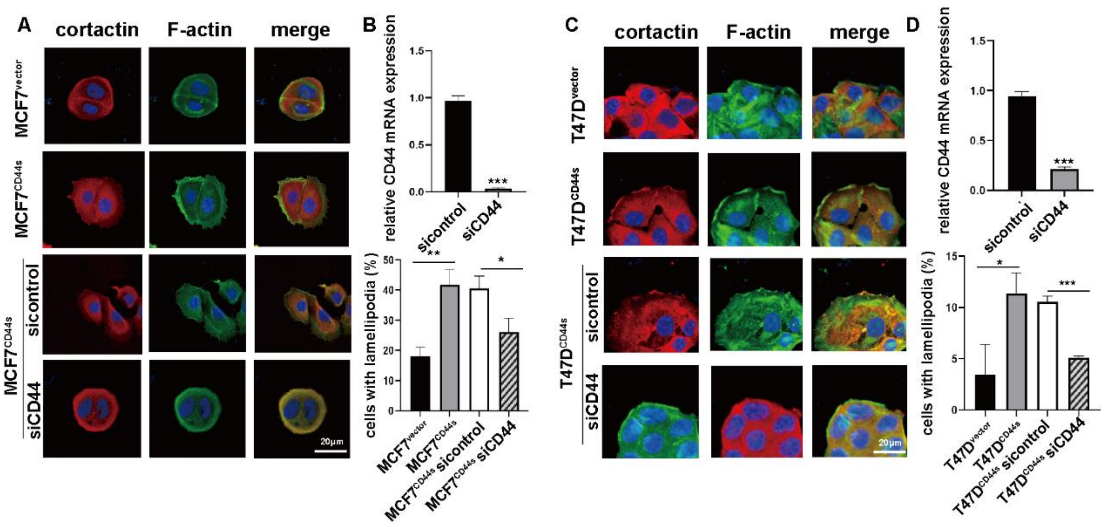

Fig. S3 loss-of-function experiment by knocking down CD44 in CD44s over-expressing MCF7 and T47D cells

(A) The distribution patterns of cortactin (red) and F-actin (green) demonstrated by immunofluorescence staining in MCF7^{vector}, MCF7^{CD44s}, MCF7^{CD44s} sicontrol and MCF7^{CD44s} siCD44 cells. The proportion of cells with lamellipodia in each group was calculated from triplicate independent experiments, means ± SD from triplicate experiments were plotted. (B) and (D) The knockdown efficiency of CD44 in MCF7^{CD44s} and T47D^{CD44s} cells, respectively. (C) The distribution patterns of cortactin (red) and F-actin (green) demonstrated by immunofluorescence staining in T47D^{vector}, T47D^{CD44s}, T47D^{CD44s} sicontrol and T47D^{CD44s} siCD44 cells. *p <0.05, **p <0.01, ***p <0.001.

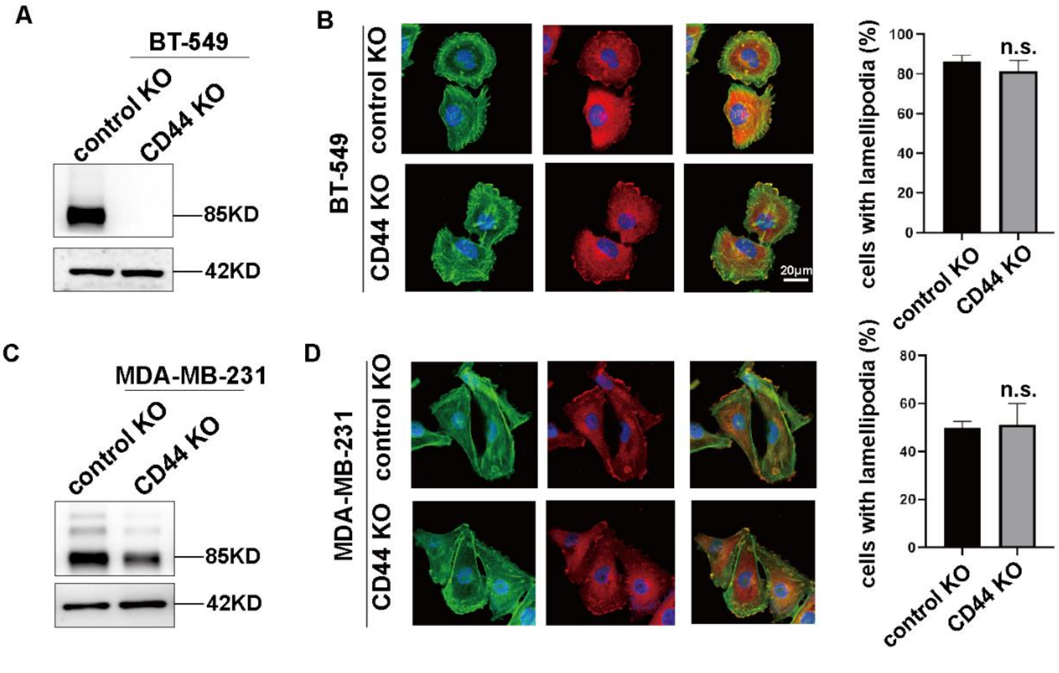

Fig. S4 The effect of CD44 in lamellipodia formation of basal-like breast cancer cells

(A) and (C) The efficiency of CD44 knockout in BT-549 and MDA-MB-231 cells. (B)

The distribution patterns of cortactin (red) and F-actin (green) demonstrated by immunofluorescence staining in BT-549 control and CD44 knockout cells. The proportion of cells with lamellipodia in BT-549 control and CD44 knockout groups were calculated from triplicate independent experiments, means ± SD from triplicate experiments were plotted. (D) The distribution patterns of cortactin (red) and F-actin (green) demonstrated by immunofluorescence staining in MDA-MB-231 control and CD44 knockout cells. n.s. indicates no significant.

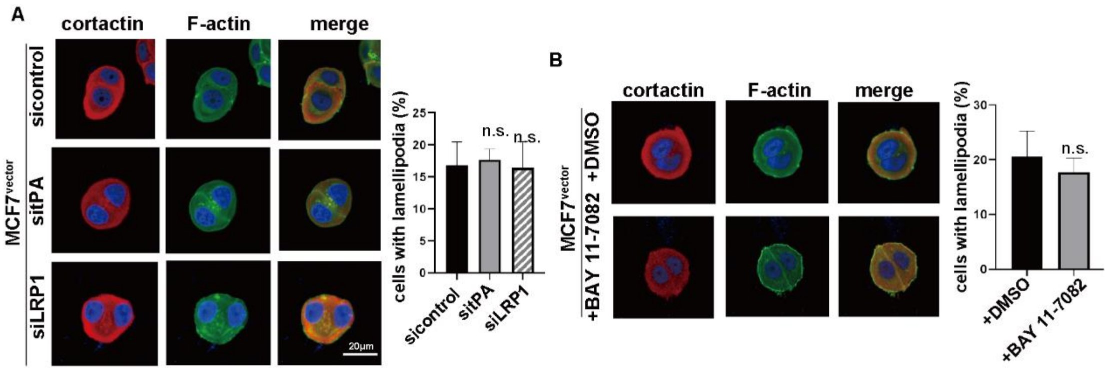

Fig. S5 The effect of tPA, LRP1, and NFKB signaling on lamellipodia formation in MCF7 ^{vector} cells

(A) and (B) The distribution patterns of cortactin (red) and F-actin (green) demonstrated by immunofluorescence staining in MCF7^{vector} sicontrol, MCF7^{vector} sitPA, MCF7^{vector} siLRP1 MCF7^{vector} DMSO, and MCF7^{vector} BAY 11-7082 cells. The proportion of cells with lamellipodia in each group was calculated from triplicate independent experiments, means ± SD from triplicate experiments were plotted. n.s. indicates no significant.

primers	sequence
ACKR3 forward	CCAAGACCACAGGCTATGACAC
ACKR3 reverse	TGGTTGTGCTGCACGAGACTGA
THBS1 forward	GCTGGAAATGTGGTGCTTGTCC
THBS2 reverse	CTCCATTGTGGTTGAAGCAGGC
RET forward	GTCCTCTTGCTCCACTTCAACG
RET reverse	CCTGGCAGTTTTCCACACAGAC
EPHA4 forward	ACCAAGCAGTGCGAGAGTTTGC
EPHA4 reverse	CTCTCTTGCCAGGCACTTTGAG
LOXL2 forward	TGACTGCAAGCACACGGAGGAT
LOXL2 reverse	TCCGAATGTCCTCCACCTGGAT
BMP7 forward	GAGTGTGCCTTCCCTCTGAACT
BMP7 reverse	AGGACGGAGATGGCATTGAGCT
SMAD7 forward	TGTCCAGATGCTGTGCCTTCCT

SMAD7 reverse	CTCGTCTTCTCCTCCCAGTATG
SLC7A5 forward	GCCACAGAAAGCCTGAGCTTGA
SLC7A5 reverse	ATGGTGAAGCCGATGCCACACT
FUT8 forward	GACAGAACTGGTTCAGCGGAGA
FUT8 reverse	GCAGTAGACCACATGATGGAGC
FGFR4 forward	AACACCGTCAAGTTCCGCTGTC
FGFR4 reverse	CATCACGAGACTCCAGTGCTGA
L1CAM forward	TCGCCCTATGTCCACTACACCT
L2CAM reverse	ATCCACAGGGTTCTTCTCTGGG
CD44 forward	CTGCCGCTTTGCAGGTGTA
CD44 reverse	CATTGTGGGCAAGGTGCTATT
tPA forward	TGGTGCTACGTCTTTAAGGCGG
tPA reverse	GCTGACCCATTCCCAAAGTAGC
GAPDH forward	GTCTCCTCTGACTTCAACAGCG
GAPDH reverse	ACCACCCTGTTGCTGTAGCCAA

supplementary table 1: The primer sequences for qPCR
